# Systematic Review of Mammalian Models for Experimental Sporotrichosis: Pathogenesis, Methodological Variables, and Ethical Considerations

**DOI:** 10.3390/ani16081226

**Published:** 2026-04-17

**Authors:** Danielly Corrêa-Moreira, Thais Guimarães Barreira, Rodolfo Castro, Cintia de Moraes Borba, Manoel Marques Evangelista Oliveira

**Affiliations:** 1 Laboratory of Taxonomy, Biochemistry and Bioprospecting of Fungi, Oswaldo Cruz Institute, Oswaldo Cruz Foundation, Rio de Janeiro 21040-900, Brazil; thais.guimaraes@ini.fiocruz.br (T.G.B.); cborba@ioc.fiocruz.br (C.d.M.B.); 2 Laboratory and Facility Multi-User, Evandro Chagas National Institute of Infectious Diseases, Oswaldo Cruz Foundation, Rio de Janeiro 21040-900, Brazil; 3 Sergio Arouca National School of Public Health, Oswaldo Cruz Foundation, Rio de Janeiro 21040-900, Brazil; rodolfo.castro@ensp.fiocruz.br; 4 Institute of Collective Health, Federal University of the State of Rio de Janeiro, Rio de Janeiro 21941-598, Brazil

**Keywords:** systematic review, experimental mammalian model, *Sporothrix* spp., sporotrichosis

## Abstract

In this review, we aimed to condense data on mammalian models of experimental infection with species of the genus *Sporothrix*, the causal agent of sporotrichosis, using the PRISMA methodology to search in three electronic databases: PubMed, Lilacs, and Web of Science. We analyzed the mammals used and the criteria that determine the course of the infection, including inoculum size and route of inoculation, the host’s immune status, and the fungal species employed, as well as information on ethical principles and criteria for determining the pathogenicity/virulence of the fungal species used, and presented a scoring system to be used in experimental infection studies in animal models alongside clinical parameters to assess the humane endpoint and provide reliable results while respecting animal welfare. Our results demonstrated that most articles described mice as mammalian models for experimental sporotrichosis. Over half of the articles cited an intermediate inoculum, ranging from 10^6^ to 10^7^ cells/mL. Subcutaneous is the inoculation route described in 27.71% of the articles, followed by intraperitoneal and intravenous routes, with 25.30% and 21.08%, respectively. Seventy-nine point five-two percent of the studies used immunocompetent models, 9.04% used immunosuppressed animals, and 10.84% included both immunocompetent and immunosuppressed animals. We also observed that *Sporothrix schenckii* was the most widely used species, considering both the entire period (1900–2024: 77.11%) and the period after the description of new species (2008–2024: 56.47%). Animal welfare conditions were poorly detailed in all articles. Only four studies reported a humane endpoint to terminate the experiment, and one presented consideration of the 3Rs (Replace, Reduce, and Refine). A few articles mentioned the most significant criteria grouped to evaluate the pathogenicity/virulence of the fungal species studied.

## Simple Summary

Sporotrichosis is an implantation mycosis caused by pathogenic species of the genus *Sporothrix*, and the use of laboratory animal models is of great importance in studying the course of the infection, providing information about its development and control. This review analyzed mammalian models for experimental sporotrichosis, focusing on factors that influence infection outcomes, including animal species, cell inoculum size, route of infection, immune status, and the *Sporothrix* species used. It is essential to mention that, despite the use of these models since the 1900s, animal welfare measures were rarely reported, with very few studies mentioning humane endpoints or ethical standards for animal research (3Rs: Replace, Reduce, Refine). It is essential to emphasize that the 3Rs principle has broad implications, particularly in promoting more ethical research. This involves attempts to replace animals with alternative models, minimize the number of animals used, and improve welfare and the methods employed. For this reason, we propose an evaluation system based on points to help researchers determine humane endpoints, aiming to improve both data quality and animal welfare in future studies.

## 1. Introduction

### 1.1. Sporotrichosis and Its Causal Agents 

Sporotrichosis is a worldwide implantation mycosis [1,2,3]. It has been reported in South America (Brazil, Colombia, Mexico, Guatemala, Peru, and Paraguay), the USA, Asia (China, Japan, and India), Australia, and Europe on rare occasions [1,2,3]. Until 2006, only *Sporothrix schenckii* was considered the causal agent of sporotrichosis; however, based on phylogenetic studies conducted by Marimon et al. [4], which described genetic variability within species, the concept changed. From then on, species such as *S. schenckii sensu stricto*, *Sporothrix brasiliensis*, *Sporothrix globosa*, *Sporothrix luriei*, *Sporothrix mexicana*, *Sporothrix pallida*, *Sporothrix chilensis*, and *Sporothrix humicola* are currently considered the causal agents of sporotrichosis [5,6,7,8,9,10,11,12,13].

*Sporothrix* species exhibit epidemiological differences in their pathogenicity, host associations, and geographical distribution [14,15,16,17,18]. All these fungi mentioned above are thermodimorphic and capable of converting themselves from the saprophytic filamentous phase, in nature or in vitro at 25–28 °C, to the yeast phase, in the mammal host or in vitro at 35–37 °C [16]. This temperature-induced transition can be a virulence factor related to the fungus’s ability to survive and acquire a parasitic form at host body temperature. Besides temperature, pH is also an important factor in the dimorphic conversion of these fungi. However, in addition to dimorphism and thermotolerance being factors related to virulence, other factors are co-responsible for the ability of species to cause damage to the host. These are cell wall proteins, melanin production, extracellular and intracellular proteinases, extracellular vesicles, lipids, and biofilm [19,20]. To investigate the pathogenic mechanisms of fungi in a physiological environment, the use of animal models is very important and an essential component of biomedical research [21,22].

### 1.2. From Their Choice of Animal Models to Ethical Principles 

The experimental models used in the study of infectious diseases must be an adequate host to reproduce in vivo infection [23]. Animal models are a key tool in the advancement of medical mycology [24]. Still, it is necessary that animal experimentation has a reasonable cost and adheres to ethical principles that define when and how to use animals in research [24,25]. The lack of standardized models in studies of fungal infection is a significant obstacle, as it leads to variability in results, making it difficult to compare studies and draw reliable conclusions [26].

In the 19th century, the use of animals in research became more frequent, initially observing only a stress reduction. In 1959, Russell and Burch described the principle of the “3 Rs”—Replace, Reduce, and Refine in animal research [27]. Over the past decade, the number of systematic reviews (SRs) in animal research has increased, aiming to promote awareness and understanding of the 3Rs concept and prevent unnecessary animal use [28,29]. In this context, the ethical evolution of animal experimentation has progressed from largely uncontrolled practices to a regulatory framework guided by the principles described above (3Rs) and institutional oversight, driven by increased public awareness, scientific debate, and advocacy groups [30].

Pain, suffering, and stress are conditions that can significantly affect research results. Based on this, humane endpoints can be defined as the cut-off or earliest indicator that can be used to avoid, stop, or limit the distress, discomfort, or potential pain and suffering in the animal [31]. In that regard, a score system becomes an essential tool in animal experimentation since its parameters help to apply the principle of the “3 Rs” to the practical environment of the laboratory.

Table 1 shows clinical parameters commonly used to determine a humane endpoint in the experimental fungal infection. Behavior, appearance, food and water intake, and clinical signs were considered based on literature data [32]. For experimental sporotrichosis, was included by our group as another parameter (Presence of lesions: sporotrichoid lesion on limbs and skin tissue, discrete inflammation and crusty lesion at and around the point inoculation) to estimate the end of the experiment the characterization of the lesions similar to that used in murine model of disseminated sporotrichosis [33], design of the animal experiments with *Leishmania* species [34], and studies of classification of feline sporotrichosis clinical presentations [35].

### 1.3. Mammalian Models for Experimental Sporotrichosis 

Mammals have been used as experimental models for a long time, due to their similarity mainly with human physiology [36]. Mammalian models, particularly rodents like mice, are vital for studying sporotrichosis because they allow observation of the effects of the fungus on the living organism, providing insights into disease progression and host response. They also help identify the virulence factors that make *Sporothrix* spp. pathogenic and help evaluate the efficacy and safety of antifungal drugs against infections caused by them. And no less importantly, models can be helpful in studies of the transmission of fungal species from animals to humans and between animals [37,38].

### 1.4. Other Animal Models

Currently, mammalian models are being replaced by non-mammalian models in fungal infections, as zebrafish, *Danio rerio* [39,40], the non-parasite nematode *Caenorhabditis elegans* [41], and insects, *Galleria mellonela* [42,43] and *Drosophila melanogaster* [44,45], among others [37]. However, the use of non-mammalian experimental models is still controversial, as it is often unknown how well these systems can predict the virulence potential of pathogens in humans or other mammals [23]. Additionally, other limitations are associated with these systems. First and foremost, the body temperature of non-mammalian hosts is generally lower than that of humans. Since many microbial virulence factors are expressed at human body temperature, this difference still constitutes an essential obstacle to using alternative models [23].

As previously mentioned, since 2006, *Sporothrix* spp. has been considered a group of pathogenic species. Over time, studies comparing different *Sporothrix* spp. using experimental mammalian models were carried out. For example, Arrilaga-Moncrieff et al. [46] published the first article comparing the different pathogenicity levels of five *Sporothrix* spp. using an immunocompetent OF-1 mice model. Recently, Corrêa-Moreira et al. [22] analyzed the clinical and anatomopathological changes in immunocompetent and immunosuppressed BALB/c mice infected with clinical and environmental isolates of seven different pathogenic species (except *S. humicola*).

However, little is known about the volume of data represented by the article that used a mammalian experimental model to reproduce this disease. Knowing these data and understanding the requirements and applications of experimental sporotrichosis using mammalian models is a valuable approach for clinical research by supporting the ethical assessment of the necessity for additional animal experiments, particularly within the ‘One Health’ framework, which underscores the interdependence of human, animal, and environmental health [28].

Based on this, the objective of this review is to describe the most widely used mammalian models in the study of sporotrichosis and the variables that directly affect the course of the infection, such as immunological status, inoculum size, site of inoculation and which pathogenic species of the genus *Sporothrix* most used in experimental studies using PRISMA (Preferred Reporting Items for Systematic Reviews and Meta-Analyses) as methodology. Lastly, information on ethical principles and criteria to assess the pathogenicity/virulence of fungal strains used in in vivo experiments on sporotrichosis are also analyzed.

## 2. Materials and Methods

PRISMA (Preferred Reporting Items for Systematic Reviews and Meta-Analyses) statement, accessed at http://www.prisma-statement.org, was used. Three electronic databases (“PubMed”, “Lilacs”, and “Web of Science”) were searched from December 2021 to 2024 for articles of any type, published from 1900 to 2024. The Medical Subjects Heading (MeSH) used were “*Sporothrix*”, “mouse”, “mice”, “mammal”, “mammalian”, “model”, “murine”, “rat”, and “hamster”. The search equations from all databases are listed in Supplementary Table S1.

Original research articles were included if they fulfilled the following criteria: (a) articles in English; (b) articles from 1900 to 2024; (c) articles that describe experimental infection of *Sporothrix* spp. in mammals. Excluded were: (a) non-peer-reviewed sources (thesis, dissertations, monographs, named as wrong publication type); (b) studies without *Sporothrix* identification (background article); (c) publications on non-mammalian models (wrong population) or naturally infected animals (wrong study design). Duplicated publications were removed after two independent reviewers screened titles and abstracts.

Readings and qualitative and quantitative analyses of the included articles to obtain data on mammalian species used in the study; inoculum size; inoculation route; immune status of the experimental model, and *Sporothrix* spp. inoculated; ethical principles (animal welfare aspects: sourcing, housing, husbandry, consideration of a humane endpoint; consideration of 3Rs—Replacement, Reduction, Refinement) and criteria for determining the pathogenicity/virulence of *Sporothrix* spp. were carried out, and results tabulated.

Data were organized for analysis and graphing using Prism GraphPad for Windows version 8 (GraphPad Software, Boston, Massachusetts USA, www.graphpad.com).

## 3. Results

**Figure 1:**
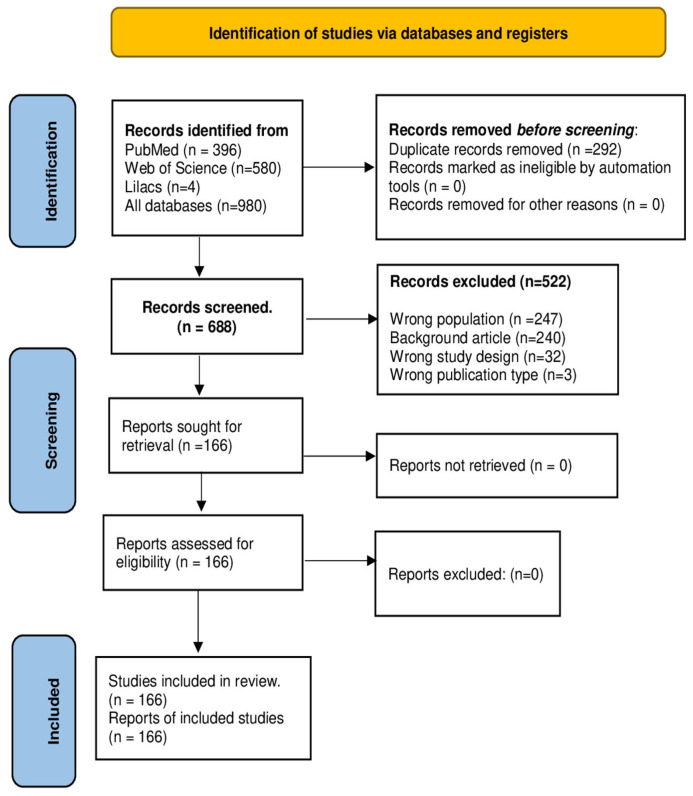
PRISMA 2020, a flow diagram of the search in experimental sporotrichosis databases.

The database survey resulted in a total of 980 records, and the selection process is shown in the PRISMA flow diagram (Figure 1). Duplicate records (292) were removed, resulting in 688 articles. After screening, 522 were excluded based on the criteria described in the methodology. Finally, 166 records on experimental sporotrichosis using mammalian models were available for analysis.

The complete list of references included in this systematic review, with information about the year of publication, authors/publication source, experimental model, inoculum size, inoculation route, immune status, *Sporothrix* species studied, and scope of the article, is shown in Supplementary Table S2.

**Figure 2:**
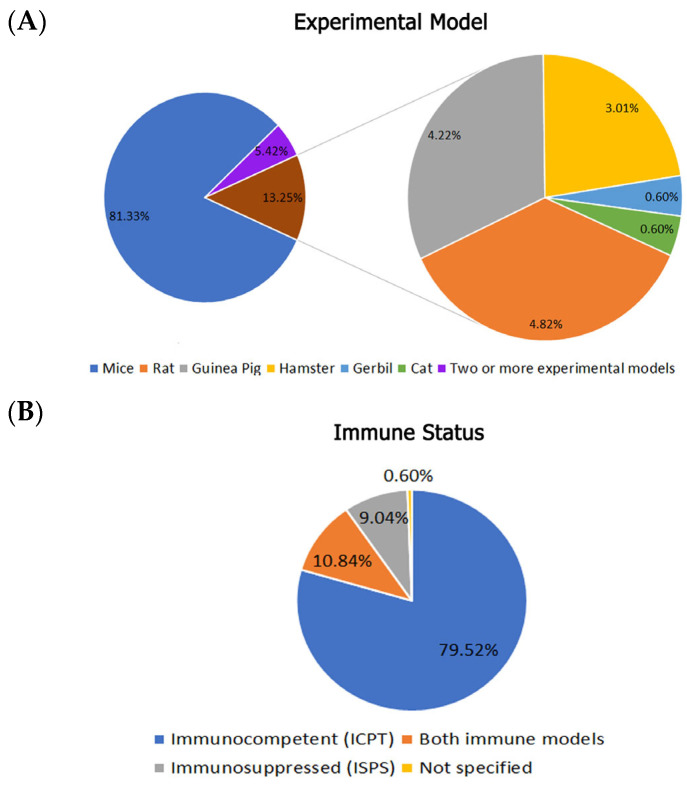
Summary of data in absolute numbers from the records obtained in the survey on experimental sporotrichosis using mammalian models in the PubMed, Lilacs, and Web of Science databases from 1900 to 2024. (**A**) Percentages of mammalian models used in the studies and (**B**) their respective immune status.

**Figure 3:**
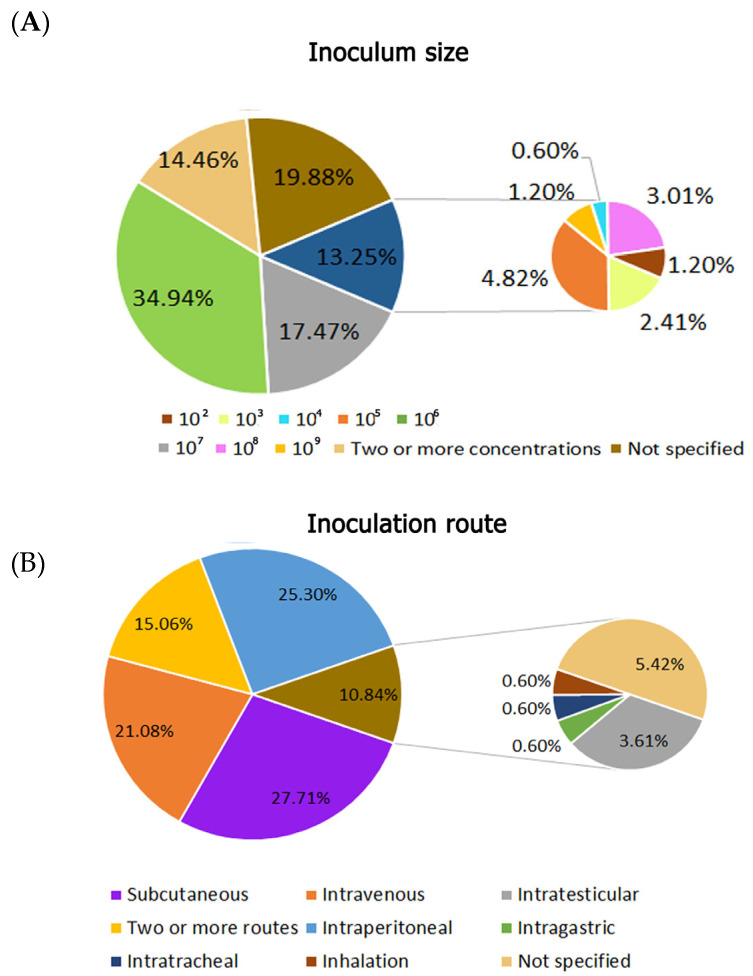
Summary of data in absolute numbers from the records obtained in the survey on experimental sporotrichosis using mammalian models in the PubMed, Lilacs, and Web of Science databases from 1900 to 2024. (**A**) Percentages of inoculum size and (**B**) their inoculation routes.

**Figure 4:**
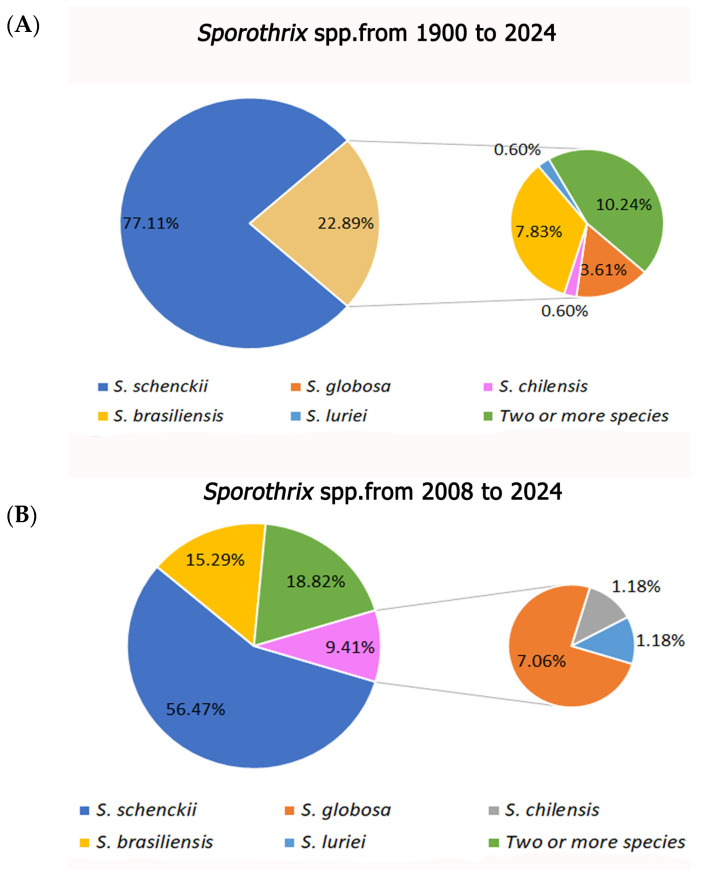
Summary of data in absolute numbers from the records obtained in the survey on experimental sporotrichosis using mammalian models in the PubMed, Lilacs, and Web of Science databases from 1900 to 2024. Percentages of *Sporothrix* inoculated species in the period from 1900 to 2024 (**A**) and from 2008 to 2024 (**B**), after new species description.

The percentages of all data referring to these records are shown in Figure 2, Figure 3 and Figure 4. Mice were the most used model (81.33%), followed by rats (4.82%) and guinea pigs (4.22%). Percentages were calculated for articles that used only a single mammalian model (Figure 2A). Genetically modified mice were first used to study sporotrichosis in 2004 and subsequently in 2008, 2014, 2017, 2019, and 2020, with an average of one article per year. The models were mostly knockout mice used in studies related to immunological factors. It is necessary to note that, although the lineage of the animals is listed in Supplementary Table S2, the percentage calculations, age, weight, and gender were not considered when conducting this survey. This represents a limitation of study and should be mandatorily considered in future studies.

Concerning immune status, 79.52% of the studies used immunocompetent (ICPT) models. Nine-point-zero-four percent used immunosuppressed (ISPS) animals, and 10.84% used both ICPT and ISPS animals (Figure 2B).

Regarding the inoculum size, it is not specified in 19.88% of the studies. However, in the articles in which it is described, it is possible to note that the preferential inoculum size varies between 10^6^ (34.94%) and 10^7^ (17.47%) cells/mL (Figure 3A). Subcutaneous is the inoculation route described in 27.71% of the articles, followed by intraperitoneal and intravenous routes, with 25.30% and 21.08%, respectively. In 9 articles (5.42%), the path of inoculation is not specified, and 15.06% of the studies used two or more routes (Figure 3B).

As for *Sporothrix* spp. inoculated, *S. schenckii* was the species reported in 77.11% of the articles, followed by studies with two or more species and *S. brasiliensis* (10.24% and 7.83%, respectively) when the entire period (1900–2024) was analyzed. This ranking remains the same, though with different percentages (56.47%, 18.82%, and 15.29%, respectively), when we focus on new species descriptions (2008–2024) (Figure 4).

The animal welfare conditions are listed in Supplementary Table S3 and were poorly detailed in all articles, with no significant differences between older and more recent articles. The highest percentages were observed in articles that used mice. Only four studies (2.51%) reported a humane endpoint to terminate the experiment (numbers 100, 162, 165, 166 in Supplementary Table S2), and one (0.62%) presented consideration of the 3Rs (number 162 in Supplementary Table S2). The same table also shows the percentage of articles that mentioned the individualized and grouped criteria for evaluating the pathogenicity/virulence of the fungal species studied. Histopathology, clinical signs, presence of fungal cells, and fungal cell recovery were the commonly used criteria in experiments using the murine model. Still in the same table, when these criteria were analyzed in groups, we found that histopathology + clinical signs + presence of fungal cells were commonly used to determine the degree of infection caused in mice.

## 4. Discussion

In this review, we demonstrated that mice were the most used model of the studies reported, followed by rats. These results demonstrate the heavy reliance on mice in research. This fact limits translational diversity, as mice, despite having significant similarities to humans in anatomy and physiology [47,48], often fail to accurately model complex human diseases and physiological processes, resulting in problems in drug translation [49]. In the quest to improve translation, researchers use crossbreeding strategies and DNA modification techniques to create animal models of human diseases. They also suggest the use of a wider variety of models, ranging from higher animals to non-animal models, for other diseases [50]. In the case of studies of sporotrichosis and its agents, mice remain the first choice and gold standard for investigations [37,47,48,51].

Of the 166 articles analyzed, 58 (34.94%) used an inoculum of 1 × 10^6^ conidia/mL. This cell concentration is the most frequently used by researchers, as it is considered an intermediate inoculum. However, the standardization of the inoculum size depends on parameters, such as resistance or susceptibility of the model lineage, route of inoculation, and immunological status [52,53,54,55,56]. For example, our group evaluating the clinical signs and immune response of mice infected with *Purpureocillium lilacinum*, a fungus considered by some authors to have relatively low virulence, which requires a high inoculum and severe immunosuppression to cause infection in an experimental host, was successful in establishing the infection with a relatively low inoculum (4.4 × 10^4^) in BALB/c mice, lineage considered more susceptible to fungal infections [57]. On the other hand, using C57BL/6 mice, a murine lineage considered more “resistant” to some pathogens, was necessary to increase inoculum size to 1 × 10^5^ conidia [58].

It is well known that the course of an infection is influenced not only by host-related factors, such as immune status, but also by fungal virulence traits, including thermotolerance, melanin production, and the secretion of extracellular enzymes [19,20]. However, for many microbes, the capacity to cause disease is highly dependent on the inoculum, and the size of the inoculum may modulate how these virulence factors are expressed [59,60].

Regarding the route of inoculation, it is known that the transmission of sporotrichosis, whether sapronotic or zoonotic, may be mainly by traumatic inoculation of the skin or mucosa with material contaminated with hyphal fragments or conidia [18]. Transmission may also occur via aerosols from infected animals that reach the ocular mucosa or, after touching the animal or fomites, individuals bring contaminated hands to their eyes. More rarely, inhalation of fungal propagules and hematogenous dissemination may occur [18]. In this review, the subcutaneous route of inoculation was the most frequently used in articles on experimental sporotrichosis (27.71%), perhaps because it is the route that better reproduces natural infection by this agent. However, there are limitations to using the subcutaneous route of infection for *Sporothrix* spp. due to the model’s inherent differences from human disease, including variations in virulence and immune response, it does not accurately reflect the complexities of human pathology [18]. The intraperitoneal and intravenous routes, even though they do not mimic natural infection, were reported as the route of choice in 25.30% and 21.08% of the articles, respectively. Generally, depending on the experimental systemic or subcutaneous sporotrichosis model, mice can be inoculated intraperitoneally or intravenously, for the first model, or subcutaneously for the second model [37]. Hohl [61] states that the inoculum should be administered in physiologically relevant infection pathways, pointing out that peritoneal and intravenous inocula are adequate to model systemic infections, which are atypical manifestations of sporotrichosis.

Furthermore, it is essential to mention that the immune mechanisms of the host-pathogen interaction can also influence the choice of the inoculation route. *Sporothrix* spp. contain various potentially antigenic molecular components (PAMPs), recognized by PRRs, expressed on host cells that trigger an effector response from cells of the innate immune response [62]. In this cell repertoire, macrophages play an essential role in defense against *Sporothrix* spp., undergoing M1 activation early and M2 in later stages of infection, but, despite that, fungi are capable of developing mechanisms of resistance to phagocytosis by these cells, delegating to neutrophils the role of eliminating pathogens. These cells efficiently bind and ingest the fungus, creating a pro-inflammatory environment that favors its clearance. Dendritic cells also phagocytize the pathogen, promoting a Th1 response in cutaneous infection and a more tolerogenic Th2-prone response in visceral sites. NK cells contribute through splenic expansion and increased systemic cytokines (TNF-α, IFN-β, IL-6). Additionally, peritoneal mast cells release early cytokines (TNF-α, IL-6) without degranulation, which may facilitate fungal dissemination and act as a negative immune regulator. Nevertheless, the intraperitoneal route of infection has some limitations in immune response studies, especially when immunosuppressed models are used [62,63].

It is important to emphasize that preparing the fungal inoculum and choosing between its morphotypes—yeast or filamentous—are crucial steps that should align with the aim of the study. The yeast form is most suitable for subcutaneous, intraperitoneal, or intravenous infection models, particularly when the objective is to study systemic dissemination or immune responses, as it closely resembles the form found in host tissues during natural infection. On the other hand, the filamentous form may be helpful in studies focusing on environmental persistence, transmission pathways, or the early stages of skin infection following traumatic implantation, as it reflects the organism’s saprophytic, ecological phase. Therefore, this form tends to be less virulent and does not always accurately represent the invasive behavior of *Sporothrix* in host tissues, which can limit its applicability in studies of pathogenesis or treatment evaluation [17].

Immunocompetent animal models were the most reported (79.52%). This result is justified since *Sporothrix* spp. is considered a “classical pathogen”, able to infect and cause disease in immunocompetent individuals, unlike opportunistic fungi, which require immunosuppression to establish the infection, as *Aspergillus* spp., *P. lilacinum*, *Cryptococcus* spp., and others [64,65,66]. The low percentage of articles (15 articles/9,04%) using immunosuppressed models does not mean the issue is less critical. Animal models with compromised immune systems require special care and housing [67], making them more laborious and expensive during selection. From 2004 (number 76 in Supplementary Table S2) onwards, studies using immunosuppressed animals began to be published, attempting to elucidate the immunological mechanisms involved in the pathogenesis of sporotrichosis. They investigated the role of nitric oxide in the susceptibility of mice to fungal infection, the involvement of TLR4 in the phagocytosis of fungal cells, and the impact of Toll-like receptors 2 and 4 on *Sporothrix* infection with promising results (numbers 82, 107, 129, 144, 150 in Supplementary Table S2).

Considering the fungal species used to infect the experimental models, *S. schenckii* was reported as the causal agent in 77.11% of the articles included in the study. However, it should be highlighted that, until 2006, *S. schenckii* was considered the only causal agent of sporotrichosis. From the first experimental sporotrichosis study published [63] until 2009, all studies point to *S. schenckii* as the infectious agent. Only in 2009, 3 years after the proposal of the *Sporothrix* complex [4], this new terminology was adopted in some experimental studies with mammalian models [46]. However, 48 articles (56.47%) were published in subsequent years without considering or mentioning the new nomenclature and referring to the agent as *S. schenckii* and not *S. schenckii stricto sensu*. Based on these data, we conclude that *Sporothrix schenckii sensu stricto* has been the most used species in experimental studies of sporotrichosis. This highlights the need for researchers to update taxonomic and nomenclatural references, as the ranking of species described before the adoption of polyphasic taxonomy has remained unchanged when analyzing the proportion of species reported in articles published from the onset of new species descriptions to the present.

Studies involving the experimental infection of two or more pathogenic *Sporothrix* species are not common (17 articles/10.24%). In these studies, the virulence of the species is generally compared, and, in this sense, it is worth mentioning only one study that showed a panel of clinical and anatomopathological alterations of mice infected with the most significant number of species currently described as pathogenic, except for *S. humicola* [22]. Likewise, neglecting the fact that *S. schenckii sensu stricto* is a cosmopolitan species causing major sapronosis in the Americas, and *S. brasiliensis* is the most virulent species within the genus and the responsible for the zoonotic transmission in Brazil, only two studies using these species in a chemically immunosuppressed murine model, since this condition may mimic the immunological status of patients undergoing the most invasive forms of sporotrichosis, were found [22,54].

Of the animal welfare aspects analyzed in this review, more than half of the articles reported the sourcing of mice used in the procedures (54.08%), which is considered vital information as it is basic data to know the origin of the animal used. For other experimental animals, the percentage of articles was even lower. For example, the first sporotrichosis study using different mammalian models was carried out by Hektoen and Perkins [68] without information on sourcing and animal welfare. The first article to mention the conditions of maintenance and feeding of the experimental animals was in 1983 (number 29 in Supplementary Table S2. It is worth remembering that concern for the well-being of experimental animals has its roots in various historical moments, but the concept of the 3Rs, described in 1959, is an important milestone. As seen in this study, it took 24 years for the scientific community to highlight this issue in scientific articles. From then on, although the information was still incipient, the conditions regarding the animals’ food and bedding began to be improved. Still, nothing was mentioned about the reduction of pain, suffering, and stress in the experimental model used.

In 1992, we verified the first manuscript (number 51 in Supplementary Table S2), which described an experimental procedure with animals approved by a specific committee and the euthanasia of animals in distress. The publication of articles reporting on the approval of experiments involving animals by ethics committees intensified (numbers 59, 67, 74, 75, 76, among others, in Supplementary Table S2). It is important to note that although the first international regulation for the use of experimental animals was in 1876 (British Cruelty to Animals Act), it took a long time for adequate controls to reach the scientific community. Given the above, an explicit description of the experimental design, including all information on the conditions to which the experimental animals are subjected, is fundamental for the credibility and validity of the studies and therefore should be described in all publications [69,70].

The insignificant percentage of the definition of humane endpoint (2.51%) and consideration of 3Rs (0.62%) seen in the articles is not exclusive to this topic. Studies related to animal models for bacteria and *Leishmania* sp. also point out the lack of descriptions of measures to safeguard animal welfare and considerations of the 3Rs [34,71]. Furthermore, trial studies with mice involving vaccination and subsequent challenges with toxins or pathogenic microorganisms are common and subject the animals to significant pain and suffering during the tests [72,73]. Conducting and analyzing studies using animals is an extensive debate that goes beyond the scientific question. Insufficient reporting of humane endpoints jeopardizes both ethical standards and data validity [74]. Humane endpoints are essential to prevent animals from unnecessarily suffering during the study. This means that everyone involved in research must know the humane endpoint’s importance and how to avoid, terminate, or alleviate animal pain during experiments [75]. Advances in legislation on the use of animals in research should be highlighted following the implementation, by different countries, of committees that regulate the use of animals in research [76] and the fundamental role of scientific journals in requesting from researchers details of the planning to conduct experiments on animals, following the highest ethical principles. However, these actions have not been sufficient because the approval of ethical protocols does not guarantee good practices in animal research, and institutional committees for the care and use of experimental animals have failed to evaluate aggressive protocols towards the animals, which could be easily refined [77,78].

These findings underscore the importance of following established guidelines for animal experimentation, such as the PREPARE [79] and ARRIVE [80] frameworks. The PREPARE guidelines focus on the planning stage of animal studies, encouraging researchers to carefully consider all aspects of experimental design, including humane endpoints, welfare conditions, and risk assessment, ultimately helping to reduce unnecessary animal use. The ARRIVE guidelines, in turn, emphasize transparent and comprehensive reporting of animal studies, aiming to improve reproducibility and facilitate critical evaluation by the scientific community. Together, these guidelines offer a complementary structure that supports ethical and rigorous research practices in alignment with the 3Rs principles (Replacement, Reduction, and Refinement).

Concerning the criteria for evaluating the pathogenicity/virulence of the fungal species studied in the articles of this review, a few articles used a greater number of grouped criteria (5.66%) to evaluate the host’s response to infection. Morbidity, mortality, body weight, clinical signs, and pathophysiology of the targeted organs are excellent criteria for evaluating infectious diseases. Researchers should consider them so that the utility of published research can be maximized, increasing the data’s reproducibility [81]. In this context, our group has been dedicated to virulence studies of fungal species in murine models in recent decades and has used well-established criteria to identify pathogenicity/virulence differences among isolates, considering animal well-being and reducing their suffering. Thus, as an example, we present a score based on our previous quantitative and qualitative assessments, which represents the different degrees of severity of changes found in an experiment (Table 2) whose content is analyzed together with clinical parameters to determine the humane endpoint shown in Table 1 to provide reliable results while respecting animal welfare. Complete and accurate information must be shared with the scientific community, and we suggest the application of these scores in all experimental designs of fungal infection using in vivo mammalian models.

## 5. Conclusions

In conclusion, this review proposes a mapping of experimental studies on sporotrichosis in in vivo mammalian models, based on articles published from 1900 to 2024. We highlight the dominance of mice as a model for disease reproduction, the lack of consideration for animal welfare with detailed animal maintenance, and the scarcity of initiative in applying the 3Rs and humane endpoints. In addition, we present a new feature, which is the scoring system for use in studies with animal models, and strongly recommend the use of standardized guidelines such as ARRIVE and PREPARE in future studies involving animal experimentation.

## Figures and Tables

**Table 1:** Clinical parameters to determine humane endpoints in the experimental fungal infection based on the literature.

Behavior	Normal	0
	Minor changes as a state of anxiety or nervous excitement	1
	Prostration and/or reduced mobility	2
	Vocalization, self-mutilation, barbering, and eye closure	3
Food and water intake	Normal	0
	Loss of up to 10% of body weight	1
	Loss of 10–15% of body weight	2
	No food and water intake or loss ≥ 20% of body weight	3
Appearance	Normal	0
	Lack of grooming	1
	Ocular and/or nasal discharges	2
	Piloerection	3
Clinical signs	Normal temperature (T), cardio frequency (C), and respiratory (R) rates	0
	Slight changes as quick breaths	1
	T ± 1 °C, C/R ↑30%	2
	T ± 2 °C, C/R ↑50%	3
Presence of lesions	No lesions at the inoculation point	0
	Discrete inflammation at the point of inoculation	1
	Crusty lesion around the point of inoculation	2
	Sporotrichoid lesion (ulcerated or non-ulcerated) on limbs and skin tissue	3
Score	Add an extra point for each parameter that received +3, and total by summing the points.	
	Total	“X”

Scoring System: 0–4 =Normal; 5–9 = Monitoring carefully and use of analgesics; 10–14 = Suffering, consider interrupting the experimentation; 15–20 = Severe pain. Euthanasia recommended. “X” = sum of the individual points for each parameter.

**Table 2:** Scoring of pathogenicity/virulence criteria used in experimental infection studies in animal models to be analyzed together with clinical parameters to determine the humane endpoints *.

Pathogenicity/Virulence Criteria	Presentation	Score
**Body weight**	= or > than the control group	0
	Loss of up to 10% of body weight	+1
	Loss of up to 10–15% of body weight	+2
	Loss of up to 20% or more of body weight	+3
**Clinical signs**	Temperature (T), cardio frequency (C), and respiratory (R) are normal; behavior and appearance are unchanged.	0
	Slight changes such as quick breaths, Lack of grooming, swelling at the site of inoculation, and adjacent tissue	+1
	Prostration and/or reduced mobility; Ocular and/or nasal discharges; T ± 1 °C, C/R ↑30%. Discrete inflammation or a crusty lesion around the point of inoculation	+2
	Vocalization, self-mutilation, barbering, eye closure, piloerection; T ± 2 °C, C/R ↑50%; sporotrichoid lesions on limbs, multiple inflammatory foci in external and internal organs	+3
**Splenic index**	1.0 (Value that expresses a unit, assigned to the weight of the control group spleens)	0
	Infected group spleen weight is 0.5 times higher than 1.0 (control group spleen weight)	+1
	Infected group spleen weight is 1.0 times higher than that 1.0 (control group spleen 1.0)	+2
	Infected group spleen weight 2.0 times or more higher than that 1.0 (control group spleen weight)	+3
**Fungal cell recovery**	None	0
	1–50 cells/spleen	+1
	51–150 cells/spleen	+2
	>150 cells/spleen	+3
**Histopathological changes**	No organ with histopathological alterations	0
	One organ with histopathological alterations	+1
	Two to three organs with histopathological alterations, or at least 1 of them damaged by 50%.	+2
	Four or more organs with histopathological alterations, or at least 1 of them damaged in 75% or more.	+3
**Presence of fungal cells/** **positive GMS**	Absent	0
	Present in 1 organ and/or less than 50 CFU per organ.	+1
	Present in 2–3 organs and/or presence of 50–99 CFU in at least 1 organ	+2
	Present in 4 or more organs and/or presence of ≥100 CFU in at least 1 organ	+3
**Survival rate**	90 to 100%	0
	50% to 89%	+1
	25% to 49%	+2
	0%	+3
**Score Total**		**“X”**

* Clinical parameters to determine humane endpoints, see Table 1. GMS = preparations of Grocott’s methenamine silver. Scoring system: 0–10—Low virulence; 11–27—Intermediate virulence; 28–42—High virulence. “X” = sum of the individual points for each parameter. ↑ = above.

## Data Availability

Not applicable.

## Supplementary Materials

The following supporting information can be downloaded at: https://www.mdpi.com/article/10.3390/ani16081226/s1. Table S1: Search strategy performed in PubMed, Lilacs, and Web of Science about experimental sporotrichosis, including research question, data of search, and medical subject heading terms. Table S2: Summary of the records on experimental sporotrichosis using mammalian models from 1900 to 2024. Table S3: Animal welfare aspects and individualized and grouped pathogenicity/virulence criteria—Percentage of articles that presented information about sourcing, housing, husbandry, the definition of humane endpoint, 3Rs consideration, and criteria to identify the virulence/pathogenicity of the fungal strains inoculated in the most used animals in the studies analyzed.
