# Supplementary material for: Systematic Review of Mammalian Models for Experimental Sporotrichosis: Pathogenesis, Methodological Variables, and Ethical Considerations
Source: Animals (Basel). 2026 Apr 17;16(8):1226. doi: 10.3390/ani16081226 (PMC13113511; doi:10.3390/ani16081226)
Supplement: Supplementary file 1 [file animals-16-01226-s001.zip › Supplementary Table 1.pdf]

Supplementary Table 1: Search strategy performed in PubMed, Lilacs, and Web of Science about experimental sporotrichosis, including research question, data of search, and medical subject heading terms.

| **My research question:** | **Experimental sporotrichosis in mammalian models** | | |
|---|---|---|---|
| **Databases** | PubMed, Lilacs, and Web of Science | | |
| **List of sources searched:** | **Date of search** | **The search strategy used, including any limits** | **Results** |
| PubMed | December 22nd, 2024 | ((Sporot*[Title/Abstract]) AND ((mice[Title/Abstract] OR mouse[Title/Abstract] OR rat[Title/Abstract] OR rabbit[Title/Abstract] OR guinea[Title/Abstract] OR swiss[Title/Abstract] OR BALB/c[Title/Abstract] OR hamster[Title/Abstract] OR mammalian[Title/Abstract] OR mammals[Title/Abstract] OR Murine[Title/Abstract] OR animal[Title/Abstract]) OR ((experimental[Title/Abstract] AND model*[Title/Abstract]) OR (animal model, experimental[MeSH Terms])))) | 396 |
| Web of Science | December 22nd, 2024 | (TS=(Sporot*) OR TI=(Sporot*) OR AB=(Sporot*)) AND (TS=(mice OR mouse OR rat OR rabbit OR guinea OR swiss OR BALB/c] OR hamster OR mammalian OR mammals OR Murine OR animal OR (experimental AND model*)) OR TI=(mice OR mouse OR rat OR rabbit OR guinea OR swiss OR BALB/c] OR hamster OR mammalian OR mammals OR Murine OR animal OR (experimental AND model*)) OR AB=(mice OR mouse OR rat OR rabbit OR guinea OR swiss OR BALB/c] OR hamster OR mammalian OR mammals OR Murine OR animal OR (experimental AND model*))) | 580 |
| LILACS | December 22nd, 2024 | Sporot$ [Words] and mice OR mouse OR rat OR rabbit OR guinea OR swiss OR BALB/c OR hamster OR mammalian OR mammals OR Murine OR animal [Words] and experimental AND model$ [Words] | 4 |
| Total of references [Identification] | December 22nd, 2024 | | 980 |
| References without duplicates [Screening] | December 22nd, 2024 | | 688 |
| Full texts articles eligible [Eligibility] | December 22nd, 2024 | | 166 |
| Studies included. [Inclusion] | December 22nd, 2024 | | 166 |
