# Supplementary material for: Systematic Review of Mammalian Models for Experimental Sporotrichosis: Pathogenesis, Methodological Variables, and Ethical Considerations
Source: Animals (Basel). 2026 Apr 17;16(8):1226. doi: 10.3390/ani16081226 (PMC13113511; doi:10.3390/ani16081226)
Supplement: Supplementary file 1 [file animals-16-01226-s001.zip › Supplementary Table 2.pdf]

Supplementary Table 2 – Summary of the records on experimental sporotrichosis using mammalian models from 1900 to 2024

	Year	Author/Publication source	Experimental Model	Inoculum size (cells/mL)	Inoculation route	Immune Status	*Sporothrix* species	Scope
1	**1900**	Hektoen and Perkins, Fifteenth Annual Meeting of the Association of American Physicians,	Rabbit/guinea pig, dog, rat, mice	NS	ip, iv, sc	ICPT	*S. schenckii*	Pathogenicity/ virulence
2	**1911**	Walker and Ritchie British Med J, July 1	Rabbit, guinea pig, rat, mice	NS	ip, iv, sc	ICPT	*S. schenckii*	Pathogenicity/ virulence
3	**1943**	Humphreys and Helmer Can J Comp Med Vet Sci, 7(7): 199-204	Rabbit, guinea pig, rat	NS	ip, sc	ICPT	*S. schenckii*	Pathogenicity/ virulence
4	**1947**	Panja, D. et al Indian Med Gaz, 82(4): 200-2	Mice, rat, rabbit, Guinea pig	NS	ip	NS	*S. schenckii , S.beurmanni,S. tropicales, S. asteroids,S. jeanselmei, S. carougeani, S. councilmani*	Pathogenicity/ virulence
5	**1949**	Neill et al. J Exp Med, 89(1): 93-106	Mice	NS	ip	ICPT	*S. schenckii*	Immunological assays
6	**1951**	Fischer and Markkanen Canad. M. A. J., 65	Mice	NS	ites	ICPT	*S. schenckii*	Pathogenicity/ virulence
7	**1959**	Hasenclever and Mitchell J Investig Dermatol, 33(3): 145-149	Swiss Mice, rabbit	2.5×10^5, 3×10^7	ip, iv	ICPT	*S. schenckii*	Immunological assays
8	**1961**	Tsubura and Schwarz Mycopathol Mycol Appl, 20(14): 55-6	Swiss Mice	2.3×10^5	iv	ICPT	*S. schenckii*	Experimental treatment
9	**1961**	Okudaira et al. Mycopathol Mycol App, 14, 284–296	Mice	1×10^6	NS	ICPT	*S. schenckii*	Experimental treatment
10	**1963**	Howard and Orr J. Bacteriol, 85: 816-821	Swiss Mice	NS	ip	ICPT	*S. schenckii*	Pathogenicity/ virulence
11	**1966**	Sethi et al. Am Rev Respir Dis, 93(3):463-4.	Mice	5.0×10^6	nas, iv	ICPT	*S. schenckii*	Pathogenicity/ virulence
12	**1968**	Beland et al. Canad. Med. Ass. J. 99: 813-816	Mice	1×10^5	ip	ICPT	*S. schenckii*	Pathogenicity/ virulence

13	**1969**	Conti-Díaz and Civilla Mycopathol Mycol App ,38: 1-6	Swiss Mice	NS	inh	ICPT	*S. schenckii*	Pathogenicity/ virulence
14	**1971**	Roberts and Larsh J Infect Dis, 124 (3): 264–269	Guinea pig	6.6×10^8, 4×10^8	icar, sc, ites	ICPT	*S. schenckii*	Immunological assays
15	**1973**	Block et al. Antimicrob Agent Chemother, 3(1): 95-98	Mice	2×10^7	iv	ICPT	*S. schenckii*	Experimental treatment
16	**1974**	Murphy et al. Infec Immun, 9(2):404-409	Guinea pig	2×10^7	sc	ICPT	*S. schenckii*	Immunological assays
17	**1975**	Shimonaka et al. Inf Immun,11(6): 1187-1194	Rabbit, Guinea pig	NS	im, sc, ip	ICPT	*S. schenckii*	Immunological assays
19	**1975**	Carrada-Bravo Ann Trop Med Parasitolo, 69(3): 267-273	Mice	NS	sc	ICPT/ISPS	*S. schenckii*	Epidemiology/ pathogenicity
19	**1976**	Feuerman et al. Sabouraudia, 14: 217-222	Mice	NS	ip, ites	ICPT	*S. schenckii*	Soil isolation/ pathogenicity
20	**1977**	Barbee et al. Am J Pathol, 86(1):281-284	Cat	NS	sc	ICPT	*S. schenckii*	Pathogenicity/ virulence
21	**1979**	Charoenvit and Taylor Infect Immun, 23(2):366-72	Hamster	5×10^3	sc	ICPT	*S. schenckii*	Pathogenicity/ Immunological assays
22	**1979**	Shiraishi et al. J Reticuloendothel Soc, 26(3):333-6	Mice	NS	NS	ISPS	*S. schenckii*	Pathogenicity/ virulence
23	**1979**	Kwon-Chung J Infec Dis, 139(4): 424-431	Swiss Mice, Guinea pig	1×10^4 , 1×10^5, 1×10^6	ip, id	ICPT	*S. schenckii*	Pathogenicity/ virulence
24	**1980**	Dixon et al. Mycopathologia, 70(3): 153-161	Mice, Hamster	1×10^6	iv	ICPT	*S. schenckii*	Pathogenicity/ virulence
25	**1980**	Hachisuka and Sasai Mycopathologia, 71(3):167-9	Guinea pig	5×10^6	sc	ICPT	*S. schenckii*	Immunological assays
26	**1981**	Hachisuka and Sasai Mycopathologia, 76: 79–82	Mice	5×10^6	sc	ICPT/ISPS	*S. schenckii*	Immunological assays
27	**1982**	Kennedy et al. Mycopathologia, 78(3):141-3	Swiss Mice	1×10^7	igas	ICPT	*S. schenckii*	Pathogenicity/ virulence
28	**1982**	Miyaji and Nishimura Mycopathologia, 80: 117-124	Mice	1×10^6	iv	ICPT/ISPS	*S. schenckii*	Pathogenicity/ virulence
29	**1983**	Dickerson et al. Inf Immun, 40(1): 417-420	Nude Mice	5×10^4, 9×10^6	ip, iv	ISPS	*S. schenckii*	Pathogenicity/ Immunological assays
30	**1983**	Fahmy et al. Mycopathologia, 82(3):175-8	Swiss Mice	1×10^8	iv	ICPT	*S. schenckii*	Experimental treatment

**31**	**1983**	Takata and Ishizaki Mycopathologia, 84: 31-39	Guinea pig	NS	sc	ICPT	*S. schenckii*	Immunological assays
**32**	**1986**	Kini et al. J Med Vet Mycol, 24: 289-295	Swiss Mice	NS	ip, ites	ICPT	*S. schenckii*	Pathogenicity/ virulence
**33**	**1986**	Schaude et al. J Med Vet Mycol, 24(4): 297-304	NMRI Mice	1×10^7, 1×10^9	sc	ISPS	*S. schenckii*	Experimental treatment
**34**	**1986**	Kazanas J F Protec, 50(11): 933-839	Swiss/ CF1 Mice	6×10^6, 2×10^7	ip, igas, po	ICPT	*S. schenckii*	Pathogenicity/ virulence
**35**	**1986**	Miyaji and Nishimura Mycopathologia, 96:143-151	Mice	2×10^6	iv	ISPS	*S. schenckii*	Immunological assays
**36**	**1987**	Van Cutsem et al. Rev Infect Dis, 9(Suppl 1): S15-32	Guinea pig	9.8×10^3	ites	ISPS	*S. schenckii*	Experimental treatment
**37**	**1987**	Kazanas et al. J F Protec,, 50(11): 933-939	Mice	1×10^6, 1×10^7	ip, igas	ICPT	*S. schenckii*	Pathogenicity/ virulence
**38**	**1988**	Hiruma et al. Arch Dermatol Res, 280(Suppl): S94-100	Mice	NS	NS	ICPT	*S. schenckii*	Pathogenicity/ virulence
**39**	**1988**	Sharma et al. Indian J Dermatol Venereol Leprol, 54: 142-147	Mice	NS	NS	ICPT	*S. schenckii*	Pathogenicity/ virulence
**40**	**1988**	Kan and Benett Antimicrob Agents Chemother, 32(11): 1619-1623	BALB/c Mice	4×10^6	iv	ICPT	*S. schenckii*	Experimental treatment
**41**	**1989**	Van Cutsem Mycoses, 32 (Suppl. 1) 14-34	Guinea pig	NS	iv/ites	ICPT	*S. schenckii*	Experimental treatment
**42**	**1989**	Shimizu et al. Mycoses, 32(9): 443-7	ICR Mice	1×10^6	ip	ICPT	*S. schenckii*	Immunological assays
**43**	**1990**	Schaude et al. J Med Vet Mycol, 28(6): 445-54	NMRI Mice, Wistar rat	1×10^5	sc	ICPT	*S. schenckii*	Experimental treatment
**44**	**1990**	González de Polanía et al. Rev Inst Med Trop São Paulo, 32(5): 319-324	Hamster	2.5×10^3	sc	ICPT	*S. schenckii*	Pathogenicity/ virulence
**45**	**1990**	Hamilton et al. Trans R Soc Trop Med Hyg, 84(5): 734-737	Mice	NS	ip	ICPT	*S. schenckii*	Pathogenicity/ virulence
**46**	**1990**	Van Cutsem Br J Clin Pract Suppl, 71:32-40	Mice	NS	NS	ISPS	*S. schenckii*	Experimental treatment
**47**	**1990**	Rajendran et al. Int J Dermatol, 29(10):716-718	Swiss Mice	NS	ip	ICPT	*S. schenckii*	Pathogenicity/ virulence
**48**	**1991**	Dixon et al. J Clin Microbiol, 29(6): 1106-1113	Mice	NS	iv	ICPT	*S. schenckii*	Pathogenicity/ virulence
**49**	**1992**	Van Cutsem Chemotherapy, 38(Suppl 1): 3-11	Guinea pig	NS	iv	ICPT	*S. schenckii*	Experimental treatment

50	**1992**	Carlos et al. Mycopathologia,117(3): 139-44	Swiss Mice	1×10^7	iv	ICPT	*S. schenckii*	Immunological assays
51	**1992**	Dixon et al. J Clin Microbiol, 30(4): 951-954	NYLAR Mice	6×10^5, 1×10^7, 2×10^8	iv	ICPT	*S. schenckii*	Pathogenicity/ virulence
52	**1992**	Shiraishi et al. Mycopathologia, 120: 15-21	BALB/c nude and normal BALB/c mice	5×10^5	iv, ites	ICPT/ISPS	*S. schenckii*	Immunological assays
53	**1993**	Restrepo-Gutierrez et al. J M Vet Mycol, 31: 411-420	BALB/c Mice	1×10^6	sc, id	ICPT	*S. schenckii*	Pathogenicity/ virulence
54	**1993**	Peng-Cheng et al. Mycopathologia, 122: 89-93	Athymic nude mice and their heterozygote littermates	1×10^8	sc	ICPT/ISPS	*S. schenckii*	Pathogenicity/ virulence
55	**1993**	De Caprilles et al. Mycopathologia 122: 129-133	Hamster	2×10^6	ites	ICPT	*S. schenckii*	Pathogenicity/ virulence
56	**1993**	Yoshiike et al. Mycopathologia, 123: 69-73	ICR Mice	1×10^8	sc	ICPT	*S. schenckii*	Immunological assays
57	**1994**	Carlos et al. Mycopathologia,127: 189-194	BALB/c and C3H/HeJ mice	1×10^6	iv	ICPT	*S. schenckii*	Immunological assays
58	**1994**	Anandi et al. Indian J Pathol Microbiol, 37(1): 97-100	Mice	NS	NS	ICPT	*S. schenckii*	Pathogenicity/ virulence
59	**1995**	Fukuzawa et al. Virchows Arch, 427: 407-414	ICR Mice	2.5×10^7	ip	ISPS	*S. schenckii*	Differential diagnosis
60	**1995**	Castrejón et al. Mycoses, 38: 373-376	Mice	NS	sc	ICPT	*S. schenckii*	Diagnosis confirmation
61	**1996**	Nakamura et al. Mycoses 39: 125-128	Mice	NS	ites	ICPT	*S. schenckii*	Diagnosis confirmation
62	**1998**	Tachibana et al. Med Mycol, 36, 21–27	BALB/c Mice	1×10^5, 1×10^6	sc, iv, ip	ICPT	*S. schenckii*	Pathogenicity/ virulence
63	**1999**	Carlos et al. Mycopathologia, 144: 9–14	Swiss Mice	1×10^6	iv	ICPT	*S. schenckii*	Pathogenicity/ virulence
64	**1999**	Fernandes et al. J Med Microbiol, 48: 195-203	BALB/c and Swiss Mice	5×10^6	iv	ICPT	*S. schenckii*	Pathogenicity/ virulence
65	**2000**	Fernandes et al. Immunology, 101: 563-569	C57BL/6 Mice	5×10^6	iv	ICPT	*S. schenckii*	Pathogenicity/ virulence
66	**2001**	Ranjana et al. Indian J Dermatol Venereol Leprol, 67(2): 86-8	Swiss Mice	NS	ip	ICPT	*S. schenckii*	Pathogenicity/ virulence
67	**2001**	da Silva et al. Microbes Infect, 3: 215–222	TS-Ab/HeTS and TR-Ab/HetS Mice	1×10^5 to 1×10^8	iv	ICPT	*S. schenckii*	Immunological assays

68	**2001**	Tachibana et al. Med Mycol, 39: 295-298	BALB/c Mice	1×10^6	iv	ICPT	*S. schenckii*	Pathogenicity/ virulence
69	**2002**	Ishizaki et al. Jpn J Med Mycol, 43: 257-260	Rat	3.5×10^5	NS	ICPT	*S. schenckii*	Pathogenicity/ virulence
70	**2002**	Mesa-Arango et al. J Clin Microbiol, 40(8):3004-3011	Taconic Mice	1×10^9	ip	ICPT	*S. schenckii*	Molecular epidemiology
71	**2003**	Randhawa et al. Indian J Med Microbiol, 21(1): 12-6	Mice	NS	ites	ICPT	*S. schenckii*	Pathogenicity/ virulence
72	**2003**	Hu et al. J Clin Microbiol, 41(4): 1414-1418	ICR Mice	1×10^6	sc	ICPT	*S. schenckii*	Molecular diagnosis
73	**2003**	Morris-Jones et al. Infec Immun, 77(7): 4026-4033	Hamster	NS	ites	ICPT	*S. schenckii*	Pathogenicity/ virulence
74	**2003**	Lima et al. Microbes Infect, 5: 933–938	BALB/c Mice	7.5×10^6	iv	ICPT	*S. schenckii*	Pathogenicity/ virulence
75	**2004**	Lima et al. Can J Microbiol, 50: 445–449	BALB/c Mice	7.5×10^6	iv	ICPT	*S. schenckii*	Pathogenicity/ virulence
76	**2004**	Kajiwara et al. Infec Immun, 72(9): 5073–5079	X-linked CGD mice	5×10^4	sc	ISPS	*S. schenckii*	Immunological assays
77	**2005**	Nascimento and Almeida FEMS Immunol Med Microbiol, 43: 241-247	BALB/c Mice	5×10^6	ip	ICPT	*S. schenckii*	Immunological assays
78	**2005**	Nobre et al. Mycopathologia, 160: 43–49	Swiss Mice	2×10^2	sc	ICPT	*S. schenckii*	Pathogenicity/ virulence
79	**2006**	Maia et al. Mycopathologia, 161: 11–19	Swiss Mice	1×10^6	ip	ICPT	*S. schenckii*	Immunological assays
80	**2006**	Kong et al. Clin Microbiol Infect, 12: 1077–1081	BALB/c Mice	2×10^6, 3×10^6	sc, iv, ip	ICPT	*S. schenckii*	Pathogenicity/ virulence
81	**2007**	Brito et al. Med Mycol, 45: 721-729	BALB/c Mice	4×10^6	sc	ICPT	*S. schenckii*	Pathogenicity/ virulence
82	**2008**	Fernandes et al. Immunology, 123: 469–479	C57BL/6 wild type and iNOS knockout mice	5×10^6	iv	ICPT/ISPS	*S. schenckii*	Immunological assays
83	**2008**	Meinerz et al. Braz J Microbiol, 39: 734-737	Wistar Rat	2×10^3	iv	ICPT	*S. schenckii*	Experimental treatment
84	**2008**	Nascimento et al. Eur J Immunol, 38: 3080–3089	BALB/c Mice	5×10^6	ip	ICPT/ISPS	*S. schenckii*	Immunological assays
85	**2008**	Nobre et al. J Mycol Med, 18: 191—197	Swiss Mice	1×10^5	sc	ICPT	*S. schenckii*	Pathogenicity/ virulence
86	**2009**	Arrillaga-Moncrieff et al. Clin Microbiol Infect,15: 651–655	OF-1 Mice	2×10^4 , 1.2×10^6, 2×10^7	iv	ICPT	*S. schenckii sensu stricto, S.*	Pathogenicity/ virulence

							brasiliensis, S. globosa, S. pallida, S. mexicana	
**87**	**2009**	Carlos et al. Mycopathologia, 168: 1–10	C3H/HeJ mice	NS	NS	ISPS	*S. schenckii*	Immunological assays
**88**	**2009**	Sassá et al. Immunology, 128: 301–309	C3H/ HeJ mice	1×10^6	ip	ISPS	*S. schenckii*	Immunological assays
**89**	**2009**	Yegneswaran et al. Inter J Dermatol, 48: 1198–1200	Swiss Mice	NS	ip, sc	ICPT	*S. schenckii*	Pathogenicity/ virulence
**90**	**2009**	Teixeira et al. Microbiology, 155: 3730–3738	C57BL/6 Mice	5×10^6	iv	ICPT	*S. schenckii*	Pathogenicity/ virulence
**91**	**2010**	Madrid et al. Microbes Infect, 12: 162-165	Wistar Rat	2×10^2	sc	ICPT	*S. schenckii*	Pathogenicity/ virulence
**92**	**2010**	Teixeira et al. Med Mycol, 48: 687–695	C57BL/6 Mice	5×10^6	iv	ICPT	*S. schenckii*	Pathogenicity/ virulence
**93**	**2011**	Lacerda et al. Mycopathologia, 171: 395–401	BALB/c Mice	5×10^6	it	ISPS	*S. schenckii*	Pathogenicity/ virulence
**94**	**2011**	Zhang et al. Mycopathologia, 172: 439–446	Kunming Mice	1×10^7	ip, sc	ISPS	*S. schenckii*	Pathogenicity/ virulence
**95**	**2011**	Franco et al. Scand J Immunol, 75: 142-146	BALB/c Mice	5×10^6	ip	ICPT	*S. schenckii*	Immunological assays
**96**	**2012**	Verdan et al. Immunobiology, 217: 788– 794	C57BL/6 Mice	1×10^7	ip	ICPT	*S. schenckii*	Immunological assays
**97**	**2012**	Almeida Front Microbiol, 3: 409	Mice	NS	NS	ICPT	*S. schenckii*	Experimental treatment
**98**	**2012**	Zhang et al. PLoS ONE, 7(12): e52514	Wistar Rat	1×10^7	sc	ICPT	*S. schenckii*	Immunological assays
**99**	**2012**	Fernández-Silva et al. Antimicrob Agents Chemother, 56(5): 2273-2277	OF-1 Mice	2×10^7	iv	ICPT	*S. schenckii sensu stricto, S. brasiliensis*	Experimental treatment
**100**	**2012**	Fernández-Silva et al. Mycopathologia, 173: 245–249	OF-1 Mice	2×10^5, 2×10^7	iv	ICPT	*S. luriei*	Pathogenicity/ virulence
**101**	**2012**	Castro et al. Mycopathologia, 174: 31-39	Wistar Rat	6×10^5, 4.5×10^6	sc, ip	ICPT	*S. schenckii*	Immunological assays
**102**	**2012**	Mendoza et al. Rev Iberoam Micol, 29(3):120–125	BALB/c Mice	3×10^2 , 6×10^2, 9×10^2, 1.2×10^3, 1.5×10^3	ip	ICPT	*S. schenckii*	Molecular diagnosis

**103**	**2012**	Sassá et al. Mycopathologia, 174: 21-30	C3H/HeJ mice	1×10^6	ip	ISPS	*S. schenckii*	Immunological assays
**104**	**2013**	Castro et al. PLoS ONE, 8(10): e75656	BALB/c Mice	1×10^7	sc	ICPT	*S. schenckii, S. brasiliensis*	Pathogenicity/ virulence
**105**	**2013**	Fernandes et al. Virulence 4(3): 241–249	BALB/c Mice	1×10^6, 5×10^6	iv	ICPT	*S. schenckii sensu stricto, S. brasiliensis, S. globosa*	Pathogenicity/ Immunological assays
**106**	**2013**	Alegranci et al. Mycopathologia, 176(1-2): 57-65	Swiss Mice	1×10^6	ip	ICPT	*S. schenckii*	Immunological assays
**107**	**2013**	Negrini et al. Immunol Invest, 42(1): 36-48	C57BL/6 wild-type and C57BL/6 TLR-2 knockout mice	1×10^8	ip	ICPT/ ISPS	*S. schenckii*	Immunological assays
**108**	**2014**	Fernández-Silva et al. Mycoses, 57(2): 121-4	OF-1 Mice	2×10^7	iv	ICPT	*S. schenckii, S. brasiliensis*	Experimental treatment
**109**	**2015**	Almeida-Paes et al. Biomed Res Int, 2015:212308	C57BL/6 Mice	5×10^7, 1×10^8	ip	ICPT	*S. schenckii, S. brasiliensis*	Pathogenicity/ virulence
**110**	**2015**	Ishida et al. Med Mycol, 53(1): 34-41	BALB/c Mice	1×10^5	iv	ICPT	*S. brasiliensis*	Experimental treatment
**111**	**2015**	de Almeida et al. Med Mycol, 53: 42-50	BALB/c Mice	5×10^6	ip	ICPT	*S. schenckii, S. brasiliensis*	Experimental treatment
**112**	**2015**	Gonçalves et al. Mycopathologia, 179: 21–30	BALB/c Mice	1×10^7	ip	ICPT	*S. schenckii*	Immunological assays
**113**	**2015**	Flores-García et al. J Chemother, 27(2): 87-93	Mongolian Gerbil	6×10^6	sc	ICPT	*S. schenckii*	Immunological assays
**114**	**2015**	Mario et al. Antimicrob Agents Chemother, 59(8): 5018-21	OF-1 Mice	2×10^7	iv	ICPT	*S. schenckii sensu stricto, S. brasiliensis*	Experimental treatment
**115**	**2015**	Ferreira et al. Immunobiology, 220(8): 985-92	BALB/c Mice	1×10^6	ip	ICPT/ISPS	*S. schenckii*	Immunological assays
**116**	**2015**	Rodrigues et al. PLoS Negl Trop Dis, 9(12): e0004190	BALB/c Mice	1×10^6, 5×10^6	iv	ICPT	*S. schenckii sensu stricto, S. brasiliensis*	Molecular diagnosis
**117**	**2016**	Maia et al. Mycopathologia, 181(3-4): 207-15	Swiss Mice	1×10^6	ip	ICPT	*S. schenckii*	Immunological assays
**118**	**2016**	Alba-Fierro et al. J Immunol Res, 2016:6525831	BALB/c and C57BL/6 Mice	5×10^6	sc	ICPT	*S. schenckii*	Immunological assays

**119**	**2016**	Portuondo et al. Immunobiology, 221(2); 300-309	BALB/c Mice	1×10^{6}	ip	ICPT	*S. schenckii*	Immunological assays
**120**	**2016**	Rodrigues et al. Fungal Biol, 120(2): 246-64	BALB/c Mice	1×10^{6}, 5×10^{6}	iv	ICPT	*S. chilensis*	Pathogenicity/ virulence
**121**	**2016**	Huang et al. Int Immunopharmacol, 34: 263-270	BALB/c Mice	1×10^{6}	sc	ICPT	*S. schenckii*	Pathogenicity/ virulence
**122**	**2016**	Cruz Choappa et al. Rev Argent Microbiol, 48(3): 196---199	CF1 Mice	1.5×10^{8}, 1.2×10^{9}	sc, ip	ICPT	*S. globosa*	Pathogenicity/ virulence
**123**	**2016**	Castro et al. Microb Pathog, 97: 94-102	Wistar Rat	6×10^{5}, 4.5×10^{6}	sc, ip	ICPT	*S. schenckii*	Immunological assays
**124**	**2016**	Batista-Duharte et al. Int Immunopharmacol, 40: 277-287	BALB/c Mice	1×10^{6}	ip	ICPT	*S. schenckii*	Experimental treatment
**125**	**2017**	de Almeida et al. Front Microbiol, 8:345	BALB/c Mice	5×10^{6}	ip	ICPT	*S. schenckii*	Immunological assays
**126**	**2017**	Oliveira et al. J Ethnopharmacol, 195: 266–274	BALB/c Mice	1×10^{7}	ip	ICPT	*S. schenckii*	Experimental treatment
**127**	**2017**	Chen et al. Sci Rep, 6: 7: 42024	BALB/c Mice	2×10^{7}	iv	ICPT	*S. globosa*	Immunological assays
**128**	**2017**	Castro et al. Microb Pathog, 107: 1-5	Rat	3×10^{6}	ip	ICPT	*S. schenckii*	Immunological assays
**129**	**2017**	Gonçalves et al. Immunology, 151: 154–166	C57BL/6 and knockout (KO) mice (NLRP3-/-, ASC-/-, caspase-1-/-)	1×10^{6}	ip	ICPT/ISPS	*S. schenckii*	Immunological assays
**130**	**2017**	Mario et al. Rev Soc Bras Med Trop, 50(4): 554-557	BALB/c Mice	2×10^{6}	ip	ICPT	*S. schenckii sensu stricto, S. brasiliensis, S. globosa, S. albicans, S. mexicana*	Immunological assays
**131**	**2017**	Della Terra et al. PLoS Negl Trop Dis, 11(8): e0005903.	BALB/c Mice	5×10^{6}	sc	ICPT	*S. brasiliensis, S. schenckii, S. globosa*	Virulence/ Immunological assays
**132**	**2017**	Portuondo et al. Vaccine, 35: 4430–4436	BALB/c Mice	1×10^{6}	ip	ICPT	*S. schenckii sensu stricto, S. brasiliensis*	Experimental treatment
**133**	**2017**	Jellmayer et al. Microb Pathog, 110: 78-84	BALB/c Mice	1×10^{6}	ip	ICPT	*S. schenckii sensu stricto*	Immunological assays

134	**2017**	Burian et al. Braz J Biol, 77(4): 848-855	Swiss mice	1×10^6	ip	ICPT	*S. schenckii*	Experimental treatment/ Immunological assays
135	**2018**	García-Lozano et al. Front Microbiol, 12:9:1275	C57BL/6 Mice	5×10^5, 10×10^6	sc	ICPT	*S. schenckii*	Immunological assays
136	**2018**	Ikeda et al. Front Microbiol 9: 2286	BALB/c Mice	1×10^7	sc	ICPT	*S. brasiliensis*	Pathogenicity/ virulence
137	**2018**	Zhang et al. Mol Med Rep, 17(1): 721-728	Kunming Mice	1×10^7	sc	ISPS	*S. schenckii*	Pathogenicity/ virulence
138	**2018**	de Almeida et al. Sci Rep, 8: 4192	BALB/c Mice	1×10^7	sc	ICPT	*S. brasiliensis*	Immunological assays
139	**2018**	Ishida et al. Med Mycol, 56: 288–296	BALB/c Mice	1×10^5	iv	ICPT	*S. brasiliensis*	Experimental treatment
140	**2018**	Batista-Duharte et al. J Fungi, 4: 64	Swiss mice	1×10^7	sc	ICPT, ISPS	*S. schenckii*	Immunotoxicity
141	**2018**	Manente et al. Med Mycol, 56(6): 711-722	BALB/c Mice	1.6×10^7	sc	ISPS	*S. schenckii*	Pathogenicity/ virulence
142	**2018**	Batista-Duharte et al. Fungal Biol, 122: 1163-1170	C57BL/6 Mice	1×10^7	sc	ICPT	*S. schenckii sensu stricto, S. brasiliensis*	Immunological assays
143	**2018**	Ferreira et al. Immunology, 155: 467–476	BALB/c Mice	1×10^6	ip	ICPT/ISPS	*S. schenckii sensu stricto*	Immunological assays
144	**2019**	Rossato et al. J Med Microbiol, 68: 87–94	C57BL/6 and TLR-2 -/- knockout mice	5×10^6	ip	ICPT/ISPS	*S. brasiliensis*	Immunological assays
145	**2019**	Oliveira et al. Mem Inst Oswaldo Cruz, 114: e190260	BALB/c Mice	1.6×10^5, 3.2×10^5	sc	ICPT	*S. brasiliensis*	Pathogenicity/ virulence
146	**2019**	Chen et al. Front Pharmacol, 10: 160	BALB/c Mice	2×10^7	iv	ICPT	*S. globosa*	Experimental treatment
147	**2019**	Lozoya-Pérez et al. Infect Drug Resist, 12: 67–85	BALB/c Mice	5×10^6	ip	ICPT	*S. schenckii*	Pathogenicity/ virulence
148	**2019**	Téllez-Martínez et al. Pharmaceutics,11(3): 144	BALB/c Mice	1×10^6	ip	ICPT	*S. schenckii sensu stricto*	Experimental treatment
149	**2019**	Li et al. Eur J Dermatol, 29(2): 160-66	BALB/c Mice	1×10^6	sc	ICPT	*S. globosa*	Experimental treatment
150	**2019**	Rossato et al. Med Mycol, 57: 489–495	C57BL/6 and TLR-4 -/- knockout mice	5×10^6	ip	ICPT/ISPS	*S. brasiliensis*	Immunological assays
151	**2019**	Najafi et al. Curr Med Mycol, 5(3): 7-12	Hamster	1.5×10^8	sc	ICPT	*S. schenckii*	Experimental treatment

**152**	**2019**	Portuondo et al.	BALB/c Mice	1×10^5, 1×10^6	iv, ip	ICPT	*S. brasiliensis*	Immunological assays
**153**	**2020**	Jiao et al. Front Immunol, 11: 469	C57BL/6 wild type and MC-deficient mice	3×10^7	sc	ICPT/ISPS	*S. schenckii*	Immunological assays
**154**	**2020**	Corrêa-Moreira et al. Future Microbiol, 15(13): 1217–1225	BALB/c Mice	3×10^6	sc	ICPT/ISPS	*S. schenckii, S. brasiliensis, S. globosa, S. mexicana, S. luriei, S. pallida, S. chilensis*	Immunological assays
**155**	**2020**	Batista-Duharte et al. Immunobiology, 225:151993	C57BL/6, DEREG Mice	1×10^7	sc	ICPT/ISPS	*S. schenckii sensu stricto*	Immunological assays
**156**	**2021**	Wang et al. Ann Dermatol, 33(1): 2021	BALB/c Mice	1×10^7	sc	ICPT	*S. globosa*	Experimental treatment
**157**	**2021**	Artunduaga Bonilla et al. Antimicrob Agents Chemother, 65(5): e02593-20	C57BL/6 Mice	5×10^6	sc	ICPT	*S. schenckii, S. brasiliensis*	Experimental treatment
**158**	**2021**	Ji et al. Dermatol Ther, 34: e14720	BALB/c Mice	1×10^7	sc, iv	ICPT	*S. schenckii*	Immunological assays
**159**	**2021**	Song et al. Braz J Microbiol, 52: 19–31	BALB/c Mice	2×10^7	ip	ICPT	*S. globosa*	Immunological assays
**160**	**2021**	Waller et al. Nat Prod Res, 35(17): 2977-2981	Wistar Rat	NS	sc	ICPT	*S. brasiliensis*	Experimental treatment
**161**	**2021**	Huang et al. BMC Microbiol, 21(1): 190	BALB/c Mice	1×10^6	sc	ICPT	*S. schenckii sensu stricto*	Immunological assays
**162**	**2021**	Corrêa-Moreira et al. Pathogens, 10: 1647	BALB/c Mice	3×10^6	sc	ICPT/ISPS	*S. schenckii, S. brasiliensis, S. globosa, S. mexicana, S. luriei, S. pallida, S. chilensis*	Pathogenicity/ virulence
**163**	**2022**	Portuondo et al. Microb Pathog, 166: 105539	BALB/c Mice	1×10^6	ip	ICPT	*S. brasiliensis*	Experimental treatment
**164**	**2023**	Munhoz et al. Med Mycol 61: myad035	BALB/cJ Mice	1×10^9	sc	ICPT	*S. brasiliensis*	Experimental treatment
**165**	**2023**	Poester et al. Mycoses, 66 (10): 898-905	mice	1×10^7	sc	ICPT	*S. brasiliensis*	Experimental treatment
**166**	**2024**	Poester at al. Curr Microbiol 81, 175	BALB/c mice	1×10^7	sc	ICPT	*S. brasiliensis*	Humane endpoint

id - intradermal; ip – intraperitoneal; iv – intravenous; nas – intranasal; inh – inhalation; it – intratracheal; ites – intratesticular; icar – intracardiac; igas – intragastric; po/par– oral/parenteral; sc– subcutaneous; NS – not specified; ICPT – immnunocompetent; ISPS - immunosuppressed.
