# Supplementary material for: Systematic Review of Mammalian Models for Experimental Sporotrichosis: Pathogenesis, Methodological Variables, and Ethical Considerations
Source: Animals (Basel). 2026 Apr 17;16(8):1226. doi: 10.3390/ani16081226 (PMC13113511; doi:10.3390/ani16081226)
Supplement: Supplementary file 1 [file animals-16-01226-s001.zip › Supplementary Table 3.pdf]

Supplementary Table 3. Animal welfare aspects and individualized and grouped pathogenicity/virulence criteria - Percentage of articles that presented information about sourcing, housing, husbandry, the definition of humane endpoint, 3Rs consideration, and criteria to identify the virulence/pathogenicity of the fungal strains inoculated in the most used animals in the studies analyzed.

| | | **Mice** | **Rats** | **Guinea pigs** |
|---|---|---|---|---|
| **Animal welfare aspects** | Sourcing | 54.08% | 1.25% | 1.25% |
| | Housing | 35.22% | 3.14% | 0% |
| | Husbandry | 31.44% | 3.14% | 0% |
| | DHE | 2.51% | 0% | 0% |
| | 3Rs | 0.62% | 0% | 0% |
| **Individualized pathogenicity/ virulence criteria** | Histopathology | 45.28% | 3.14% | 1.25% |
| | Clinical signs | 37.73% | 4.40% | 1.25% |
| | Weight loss | 7.54% | 0% | 0% |
| | Presence of fungal cells | 43.39% | 3.77% | 1.25% |
| | Fungal cell recovery | 45.28% | 0% | 0% |
| | Survival rate | 25.78% | 0% | 0% |
| **Grouped pathogenicity/ virulence criteria** | FCR + SR | 5.03% | 0% | 0% |
| | H + CS + PFC | 8.17% | 2.51% | 0.62% |
| | H + CS + PFC + FCR + SR | 5.66% | 0% | 0% |
| | H + CS + WL + PFC + FCR + SR | 5.66% | 0% | 0% |

Sourcing – Commercial and non-commercial breeders; Housing – maintenance in cages or microisolators, individually or in groups; Husbandry – animal care, including feeding and maintenance; DHE – definition of humane endpoints to terminate study participation; 3Rs - consideration of replacement, reduction, and refinement.

FCR = Fungal cells recovery; SR = Survival rate; H = Histopathology; CS = Clinical signs; PFC = Presence of fungal cells; WL = Weight loss.
